# Personality Factors as Predictors in Burnout Level Changes for Surgical Area Nurses

**DOI:** 10.3390/brainsci12111481

**Published:** 2022-11-01

**Authors:** Almudena Velando-Soriano, Gustavo R. Cañadas, Carolina S. Monsalve-Reyes, José L. Romero-Béjar, Francisco Javier Esquivel, Emilia I. De la Fuente-Solana, Guillermo Arturo Cañadas-De la Fuente

**Affiliations:** 1 Andalusian Health Service, San Cecilio Clinical University Hospital, 18071 Granada, Spain; 2 Department of Didactic of Mathematics, Faculty of Education Science, University of Granada, 18071 Granada, Spain; 3 Departamento de Ciencias Sociales, Universidad Católica de La Santísima Concepción, Concepción 4030000, Chile; 4 Department of Statistics and Operations Research, University of Granada, Fuentenueva, 18071 Granada, Spain; 5 Instituto de Investigación Biosanitaria (ibs. GRANADA), 18012 Granada, Spain; 6 Institute of Mathematics of the University of Granada (IMAG), Ventanilla 11, 18001 Granada, Spain; 7 Brain, Mind and Behaviour Research Center (CIMCYC), University of Granada, 18071 Granada, Spain; 8 Faculty of Health Sciences, University of Granada, 18016 Granada, Spain

**Keywords:** burnout, depression, logistic regression, nurses, personality risk factors, surgical area

## Abstract

Surgical area nurses provide comprehensive care to patients throughout the surgical process. Increases in life expectancy lead to the appearance and development of diseases, translating into an increase in the number of necessary interventions. Increases in the workload can be another risk factor for the development of burnout in professionals in this area. Knowledge of psychological and personality-related variables provides relevant information of level changes in the dimensions of burnout syndrome. Three logistic regression models, based on a cross-sectional study with 214 nurses working in the surgical area in the Andalusian Health Service, Spain, were built for each dimension. These models included different variables related to depression and personality, with some being significant at the population level and consequently true risk or protection factors. Friendliness, responsibility and extraversion are protection factors for the personal accomplishment dimension, whilst neuroticism is a risk factor for this dimension. Friendliness is also a protection factor for depersonalization, whilst depression is a risk factor. Finally, neuroticism, responsibility and depression are risk factors for the emotional exhaustion dimension of burnout. These findings provide relevant information that makes anticipation of this syndrome in this group easier.

## 1. Introduction

Emotional exhaustion and the loss of motivation and commitment caused by work was the first definition of burnout, given by Freudenberger in the 1970s [1]. Subsequently, Maslach and Jackson (1981) defined it as the consequence of chronic stress in work frameworks of the healthcare type [2]. To date, this is the most accepted definition of the syndrome, which is three-dimensional in nature. Burnout is considered to have three dimensions: emotional exhaustion (EE), depersonalization (DP) and low personal accomplishment (PA). EE is the physical and emotional exhaustion that professionals suffer; DP refers to the development of negative and cynical attitudes towards others and towards oneself; and PA is the adoption of a negative self-concept in relation to job performance [3]. Although there are many other scales for its evaluation, the most widely used is the Maslach Burnout Inventory (MBI), also developed in 1981. This psychological evaluation scale comprises three subscales, one for each burnout dimension. Nine items evaluate EE, five DP and eight low PA. The MBI quantifies the level of burnout as low, medium or high and has been adapted for use in different groups and in different languages [4].

As already mentioned, burnout affects the work performance of professionals and can also lead to physical and mental health problems [5], which makes it a highly relevant and topical problem. Interest in the study of this syndrome has been increasing exponentially, as reflected in the number of studies published on the subject since its recognition in the medical sphere. In 2019, it was included in the International Statistical Classification of Diseases and Related Health Problems (ICD-11) of the World Health Organization (WHO), which defines burnout syndrome as: “the result of chronic stress in the workplace that has not been managed successfully” [6].

Health professionals have constant contact with people, with nurses being a group that is especially vulnerable to the appearance and development of burnout. For this reason, they may suffer from health problems such as headaches, insomnia, musculoskeletal pain, irritability or drug use [7]. This has repercussions on the institutions where they carry out their work, as it leads to an increase in work absenteeism and also an increase in complaints from relatives of patients [8,9]. Nurses and burnout have been the object of study on multiple occasions, with a particular focus on analyzing the possible relationship between the level of burnout experienced and different variables. The sociodemographic [10,11,12] and occupational [12,13] characteristics are undoubtedly the ones that most influence the disease. One of the occupational variables that has been studied in different studies is the unit or service in which the nurses carry out their work. Just as the patients treated and the situations they face are different in each service, so are the results obtained regarding burnout [14,15].

Likewise, the number of studies that analyze the relationship between burnout and the psychological variables of nurses is lower [16]. Some of them have studied the five personality factors jointly with the appearance and development of burnout. The five-factor model was created by Costa and McCrae, in which they identified the five major dimensions of personality: neuroticism (Nm), the level of emotional instability; extraversion (Ex), the level of energy and sociability; openness (Op), the level of intellectual curiosity and aesthetic sensitivity; agreeableness (Fr), interpersonal tendencies to approach or reject others; and responsibility (Ry), self-control [17].

Surgical nurses perform a highly specialized job that requires specific training. Contact with the patient differs from that which may be had in other services, being responsible for their care and safety from the time they are received in the operating room until they are transferred to the resuscitation units. The course of events in the operating room can change in an instant, requiring nurses to be able to react to various complications [18,19,20] and subjecting them to great amounts of stress [21].

There are few studies on burnout in surgical nurses, and none were found specifically on the relationship between the psychological variables of nurses in the surgical area and the development of burnout. Therefore, this study aimed at identifying risk and/or protection factors related to personality and depression variables involved in changes in the level of severity for each dimension of burnout syndrome for nurses in the surgical area, and quantifying the effect of these factors on prognosis at the different levels of each dimension.

## 2. Materials and Methods

### 2.1. Sample

The sample comprised 214 nurses working in the surgical area at the Andalusian Health Service. The mean age of the subjects was 43.98 years (SD = 9.01), and 68.2% of them were female. The mean duration in the profession was 246.25 ± 116.19 months, and the mean time they had spent in their current position was 136.38 ± 116.66 months. A total of 40.7% of the nurses worked on a fixed morning shift and 59.3% on a rotating shift. In total, 24.1% of all nurses were on duty. 

### 2.2. Variables and Instruments

The sociodemographic variables age and sex, the dimensions of burnout (EE, DP and PA) and the personality factors of the Spanish version of the NEO Five-Factor Inventory (NEO-FFI) were obtained by means of an ad hoc questionnaire [22,23].

The Spanish-language version of the Maslach Burnout Inventory (MBI) was used to measure the three dimensions of burnout [24]. This version of the MBI consists of 22 items, 9 for EE, 5 for DP and 8 for PA. This is a 7-point Likert scale ranging from 0 (never) to 6 (every day). The reliability of the questionnaire was calculated for each dimension: EE Cronbach’s alpha: 0.887; DP Cronbach’s alpha: 0.692; PA Cronbach’s alpha: 0.842. The values proposed in the MBI handbook were used for the classification of burnout level as low, medium or high [3]. The NEO-FFI consists of 60 items in total, with each personality variable consisting of 12 items with a Likert scale of five points each. The total score of each one is obtained by adding them together. With this scale, we assessed neuroticism (N), extraversion (E), friendliness (F), responsibility (R) and openness (O). Reliability coefficients were calculated for each of the factors: N (α = 0.762), E (α = 0.775), F (α = 0.644), R (α = 0.787) and O (α = 0.718).

The Clinical-Educational Questionnaire: Anxiety and Depression (CECAD) [25] was used to measure anxiety and depression. It is a Likert-type scale with a total of 50 5-point items (26 items to assess depression and 19 items to assess anxiety). The calculated confidence level for anxiety (α = 0.905) and depression (α = 0.924) of the CECAD was calculated.

### 2.3. The procedure

A cross-sectional study was performed with a sample of 214 surgical area nurses in Andalusia (Spain). All those who agreed to participate and who were working in the surgical area at that time were included, regardless of seniority in the position.

### 2.4. Ethics

A total of 300 physical surveys were distributed in the surgical services of the different hospitals by the supervisors. A total of 237 completed surveys were received and, finally, 214 correctly completed surveys were selected. Participation in the study was voluntary, individual and anonymous. The time needed to fill out the questionnaire was approximately 45 minutes. All participants gave their written informed consent once they received information on the study. The study complied at all times with the corresponding sections of the Declaration of Helsinki [26] and received a favorable ethical opinion from the PEIBA (Biomedical Research Ethics Portal in Andalusia) with reference number 1961-N-21. All data were processed in accordance with Organic Law 3/2018 on data protection and the guarantee of digital rights [27].

### 2.5. Statistical methods

A descriptive analysis for quantitative (mean, standard deviation, maximum and minimum) and qualitative (frequencies and percentages) variables was performed. An ordinal logistic regression model was fitted [28,29] for each dimension of burnout, based on personality and depression variables. This model was fitted stepwise using forward–backward selection. Likelihood ratio test and Stukel’s chi-squared test were obtained to assess the goodness of fit. Wald’s test was used to analyze the statistical significance of the variables within the model. The prognosis ratios were obtained for the explanatory variables considered as significant at the population level. R Statistical Computing Software (version 4.3.1, https://www.r-project.org (accessed on accessed on 4 August 2022)) was used for the statistical analyses.

## 3. Results

### 3.1. Sample Description

Table 1 shows the mean and standard deviation of the personality and depression variables.

**Figure 1:**
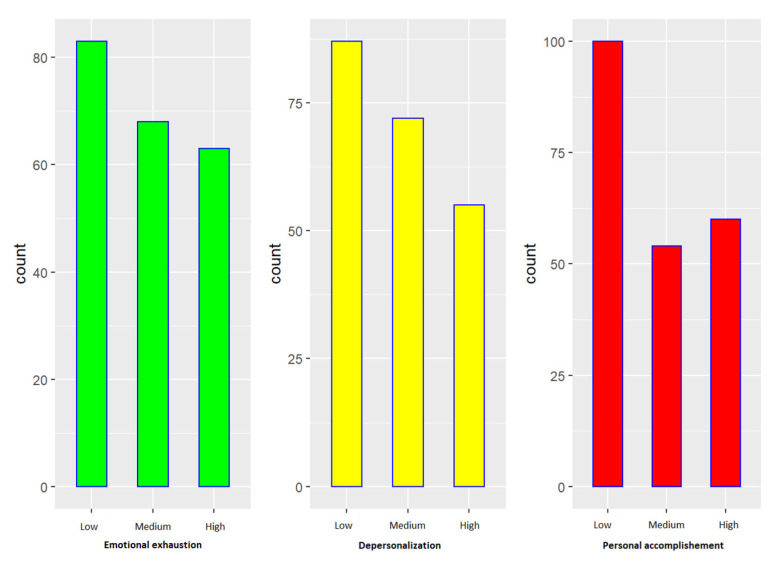
Distribution of burnout dimensions.

The MBI indications categorize each dimension of burnout syndrome as low, medium or high. Table 2 shows a descriptive analysis of the levels for each dimension of burnout in this sample, and Figure 1 shows the distribution of each level’s prevalence for each dimension.

### 3.2. Model Adjustment for Each Dimension of Burnout

This section provides a summary of the different variables included within each one of the ordinal logistic regression models fitted for each dimension of burnout syndrome.Table 3,Table 4 and Table 5 below show this summary.

The Stukel test for each model was *X*^2^ (2,N = 214) = 1.15, *p* = 0.562 for EE; *X*^2^ (2,N = 214) = 0.55, *p* = 0.757 for DP and *X*^2^ (2,N = 214) = 2.19, *p* = 0.334 for PA. According to these results, all the models produced significant goodness of fit at the population level. 

According to Wald test results (see Table 3, Table 4 and Table 5), the variable depression (D) is statistically significant at the population level (*p* < 0.001) for the models related to EE and DP dimensions. Neuroticism (N) and responsibility (R) are statistically significant (*p* < 0.05) for the models referred to EE and PA. Finally, friendliness (F) is statistically significant for the models involved in level changes in the DP and PA dimensions of burnout (*p* < 0.05), whilst E (E) is only involved in level changes in PA dimension. Odds ratios (ORs) (see Table 3, Table 4 and Table 5) for the statistically significant variables are measures of their strength for prognosis in an increasing or decreasing severity level in each of the dimensions. In fact, if OR < 1, the variable can be considered as a protection factor, whilst for OR > 1, this variable will be considered as a risk factor of worsening for the dimension of burnout under analysis. In this sense, F is a protection factor for the DP and PA dimensions, R and E are protection factors for the PA dimension, and all the other statistically significant variables are risk factors for the dimension in which they are included. As all the statistically significant variables in the models were discrete variables, a one-unit increase in their values (OR close to one) was not considered significant. In this sense, an increase of five units in D indicates that the odds ratio of moving to a high level of EE (concurrently with a high level of DP) was 5.29 (and 5.30, respectively) times greater than if D did not increase. The same occurred with N in EE and PA. Thus, with an increase of five units in N, the odds ratio of passing to high levels of EE was 5.56 times greater and 5.24 greater for PA. An increase of five units in the value of R produced 5.31 and 4.24 times greater odds ratios of changing to high levels of EE and moving to a low level of PA greater, respectively. With regard to F, an increase of five units produced 4.61 and 4.34 times greater odds ratios of moving to low levels of DP and PA, respectively. Finally, the odds ratio of moving to low levels of the PA dimension was 4.67 times greater after a five-unit increase in the values of E.

## 4. Discussion

The goal of this work was to establish risk and/or protection factors related to depression and personality variables involved in changes in the severity level for the different dimensions of burnout syndrome for nurses in the surgical area and quantify the effect of these factors on prognosis at different levels. Regarding the first part of this goal and the personality- and depression-related variables, three models were obtained. These models provide relevant information in relation to changes in the severity level in each one of the three dimensions of burnout syndrome. The models included different variables related to the personality and depression factors. The model related to EE involved the variables neuroticism, F, responsibility, extraversion and depression. The variables F and depression were the only variables included in the model that assessed changes in the severity level in the dimension of DP. Finally, neuroticism, F, responsibility and extraversion were included in the model for prognosis of a worsening level in the dimension of PA.

Regarding the second part of the objective of this work, the results show that different variables were real risk factors because high values were associated with situations of greater seriousness in the three dimensions of burnout, whilst other variables were protection factors since high values were associated with situations of lower burnout seriousness in the corresponding dimension of this syndrome. Indeed, F, responsibility and extraversion are protection factors for the PA dimension, whilst N is a risk factor for this dimension. F is also a protection factor for DP, whilst depression is a risk factor. Finally, neuroticism, responsibility and depression are risk factors for the EE dimension of burnout. 

The relationship between N and EE that has been found in this study coincides with other studies carried out in managing nurses, oncology service nurses or in nurses in the area of primary health care [30,31,32]. N is characterized by emotional instability where the subject is predisposed to interpret events in a negative way. This pessimistic tendency towards a negative view of reality can lead to EE and a lack of resources, as well as greater attention being paid to the affective state than to the work to be performed [18,33].

Depression is related to EE and DP. The values of these dimensions increase as symptoms related to depression increase. In fact, there have been cases of nurses in which it was related to the three dimensions of burnout [32]. It is important to highlight the difference between burnout and depression, an issue that generates wide debate. Both disorders are closely related but differ mainly with respect to the feeling of superiority, i.e., a depressed person experiences fewer feelings of superiority compared to a nondepressed person [34,35].

The specificity of the surgical service and the working conditions (available personnel, number of operating rooms, urgent operations, etc.) suppose an added stress that affects the adaptation and functioning of the nurses. Responsibility is important because those nurses who have this characteristic in their personality tend to make fewer mistakes and not postpone the nursing care that patients need. However, in this study, relationships between EE and responsibility were observed. This relationship may be due to the perception of excessive responsibility or feeling that the nursing profession is inherently good work, which influences their motivation and work. For this reason, it would be necessary to create a work environment where the empowerment of the nurse is encouraged and real work objectives are set, guaranteeing adequacy of resources and schedules. [19,33,36,37].

The mental health of nurses should be an object of improvement for all health authorities, and many authors confirm this [38]. An increase in burnout among workers leads to worsening of the care they give to users. This in turn decreases the quality of care and could translate into an increase in the morbidity and mortality of patients [39,40]. Recognizing the psychological risk profile of burnout in these nurses could be a useful tool to develop prevention and intervention strategies for the syndrome. These strategies are necessary to improve the health of workers, so they should be a priority for managers and politicians. Therefore, the study of psychological variables is relevant in ensuring measures are implemented as efficiently as possible. These findings provide relevant information aiding in the improved anticipation of this syndrome in this group of nurses.

### 4.1. Clinical Implications

Surgical area nurses represent a vulnerable group when it comes to developing burnout. Work environment, workload and occupational stress are risk factors for the development of various psychological disorders [41]. Knowledge of the psychological profiles of nurses prone to developing burnout could help managers anticipate the problem and provide early intervention by promoting active coping strategies, problem-solving skills training and psychological empowerment therapies [42,43,44]. Improving the mental health of nurses will allow them to be more efficient in their work, and therefore the quality of person-centered care will increase [45].

### 4.2. Study Limitations

This analysis presents some limitations. The study was carried out with nurses from hospitals belonging to the public health system of Andalusia (Spain), and may not be extrapolated to health systems in other countries that may differ in their organization and ways of working. Finally, as it was a cross-sectional study, causal relationships cannot be established. To study these relationships, a prospective longitudinal study could be carried out in the future, analyzing the evolution of burnout in nurses in the surgical area.

## 5. Conclusions

Surgical area nurses constitute a risk group for burnout syndrome due to the particular nature of the work that they perform. More than half of the nurses who participated in the study had medium or high levels in the three dimensions of burnout, around 60% in the cases of EE and DP. All psychological variables represent protective factors for one, several or all the dimensions of burnout. Managers should consider these data to meet the needs of professionals, identify risk profiles and promote coping strategies to prevent or mitigate burnout.

## Figures and Tables

**Table 1:** Descriptive analysis.

Variable (*N* = 214)	Mean	SD
N	28.11	7.26
F	45.78	6.22
R	47.70	6.12
E	42.64	7.03
O	38.89	7.00
D	51.05	14.69

Note: N = neuroticism, F = friendliness, R = responsibility, E = extraversion, O = openness, SD = standard deviation, D = depression.

**Table 2:** Descriptive analysis of the three levels of burnout for each dimension.

Levels	EE			DP			PA		
	Low	Medium	High	Low	Medium	High	Low	Medium	High
**%**	38.8	31.8	29.4	40.7	33.6	25.7	46.7	25.2	28.1
**Mean (SD)**	16.69 (11.49)			6.25 (5.57)			36.70 (8.42)		

Note: EE = emotional exhaustion, DP = depersonalization, PA = personal accomplishment, SD = standard deviation.

**Table 3:** Emotional exhaustion (EE) ${\hat{L}}_{s}\left(N,F,R,E,D\right)={\hat{B}}_{0}+{\hat{B}}_{N}N+{\hat{B}}_{F}F+{\hat{B}}_{R}R+{\hat{B}}_{E}E+{\hat{B}}_{D}D;s=1,2$.

*Predictor*	*B*	*SD*	*Wald*	*p*	*Odds*	*CI for 95% Odds*	
						*Lower*	*Upper*
*N*	0.106	0.028	3.844	<0.001	1.112	1.054	1.175
*F*	−0.042	0.027	−1.539	0.123	0.959	0.908	1.011
*R*	0.060	0.029	2.036	0.042	1.061	1.003	1.123
*E*	−0.034	0.024	−1.417	0.157	0.967	0.923	1.013
*D*	0.056	0.015	3.839	<0.001	1.057	1.028	1.089

*Note*: EE = emotional exhaustion, N = neuroticism, F = friendliness, R = responsibility, E = extraversion, D = depression, B = parameter, SD = standard deviation, Wald = Wald statistic, *p* = *p*-value, Odds = odds ratio, CI = confidence interval, Lower = CI lower limit, Upper = CI upper limit.

**Table 4:** Depersonalization (DP) ${\hat{L}}_{s}\left(F,D\right)={\hat{B}}_{0}+{\hat{B}}_{F}F+{\hat{B}}_{D}D;s=1,2$.

*Predictor*	*B*	*SD*	*Wald*	*p*	*Odds*	*CI for 95% Odds*	
						*Lower*	*Upper*
*F*	−0.079	0.025	−3.167	0.001	0.924	0.879	0.969
*D*	0.058	0.011	5.234	<0.001	1.056	1.038	1.084

*Note*: DP = depersonalization, F = friendliness, D = depression, B = parameter, SD = standard deviation, Wald = Wald statistic, p = p-value, Odds = odds ratio, CI = confidence interval, Lower = CI lower limit, Upper = CI upper limit.

**Table 5:** Personal accomplishment (PA) ${\hat{L}}_{s}\left(N,F,R,E\right)={\hat{B}}_{0}+{\hat{B}}_{N}N+{\hat{B}}_{F}F+{\hat{B}}_{R}R+{\hat{B}}_{E}E;s=1,2$.

*Predictor*	*B*	*SD*	*Wald*	*p*	*Odds*	*CI for 95% Odds*	
						*Lower*	*Upper*
*N*	0.047	0.024	1.985	0.047	1.048	1.001	1.098
*F*	−0.141	0.031	−4.544	<0.001	0.868	0.815	0.921
*R*	−0.165	0.033	−4.965	<0.001	0.848	0.793	0.903
*E*	−0.069	0.024	−2.869	0.004	0.934	0.890	0.978

*Note*: PA = personal accomplishment, N = neuroticism, F = friendliness, R = responsibility, E = extraversion, B = estimated parameter, SD = standard deviation, Wald = Wald statistic, p = p-value, Odds = odds ratio, CI = confidence interval, Lower = CI lower limit, Upper = CI upper limit.

## Data Availability

Not applicable.
